# Effect of the Freeze‐Drying Preservation Process on Some Quality Attributes of Pork Meat (*Longissimus thoracis*)

**DOI:** 10.1002/fsn3.4598

**Published:** 2024-11-11

**Authors:** Jonathan Coria‐Hernández, Rosalía Meléndez‐Pérez

**Affiliations:** ^1^ Laboratory 13 Thermal and Structural Analysis of Materials and Foods National Autonomous University of Mexico‐Superior Studies Multidisciplinary Research Unit, Faculty at Cuautitlan (UNAM–FESC), Campus 4 Cuautitlan Izcalli Mexico

**Keywords:** freeze‐dried, pork meat, preservation, quality

## Abstract

Meat preservation processes have been widely studied over time, especially those related to low temperatures (freezing and freeze‐drying); however, there is very little research that directly relates the effect of these processes on the structure of meat—and the main meat proteins—and how these changes affect some attributes of the final quality. Pork loin meat (*Longissimus thoracis*) was used, which was frozen–thawed and freeze‐dried‐rehydrated to subsequently evaluate changes in its chemical composition and physicochemical parameters such as water activity (*a*_w_), pH, and water‐holding capacity. Physical aspects such as color profile, surface myoglobin fraction, shear force, and histological sections were also evaluated, along with thermal analysis by modulated differential scanning calorimetry. The data obtained were analyzed through different statistical techniques. It was found that the freeze‐drying process significantly modifies the interactions of water with the rest of the meat components (*p* < 0.05), promoting differences concerning samples that were only frozen, allowing us to establish the importance of water and its associations with each other and with proteins on their effect on meat preservation processes at low temperatures.

## Graphical abstract

Freeze‐drying is a preservation method that generates minimal changes in the meat structure. Freeze‐drying is an excellent alternative to meat preservation.

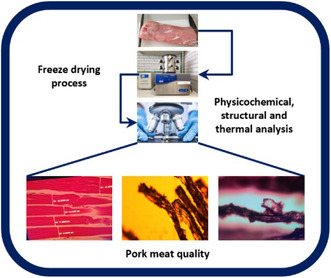

## 1 Introduction

In general, food preservation involves controlling or preventing the growth of microorganisms such as bacteria, fungi, and yeast; in some cases, this can be achieved by adding some favorable microorganisms. In this sense, conservation processes also manage to delay the oxidation of lipids, which can cause rancidity and some biochemical reactions (mainly enzymatic) (Piechnik et al. 2024; Shen et al. 2024).

In all cases, food preservation processes involve a series of different methods, each of which is an independent technique, maintaining sanitary or microbiological quality, sensory quality (color, flavor, texture), and nutritional quality as an integral part. This is why conservation prolongs the duration of safe storage of products by limiting deterioration reactions and, therefore, creates their availability throughout the season by compensating for deficiencies, as well as facilitating transportation (Ndoye et al. 2007).

The industry strives to store and transport food for long times and distances without any detrimental effect on food quality. The quality and safety of food products are also the two factors that most influence the choices made by consumers today. This is why advanced processing technologies are the key to maintaining quality, adding value, and increasing exports in a wide range of food products (Kaur et al. 2024).

In the food industry, interest in the freeze‐drying process at a commercial level arises from obtaining products with the so‐called “superior quality” compared to that obtained by conventionally dehydrated foods (hot air drying, solar drying, among others). Freeze‐drying processing is performed at low temperatures, thereby preserving flavor, color, and appearance and minimizing thermal damage to heat‐sensitive nutrients (Bailes, Meyer, and Piltz 2022; Bettaieb et al. 2024; Chen et al. 2023; Elbrink et al. 2023). Likewise, since the entire process occurs in a solid state, important structural changes in the materials, such as cell contraction and collapse, are largely avoided. However, this type of conservation process is an economically expensive method and is only viable in the case of products with high added value where the quality of the product justifies the higher production cost (Ishwarya, Anandharamakrishnan, and Stapley 2015; Meléndez‐Pérez et al. 2022).

In general, it must be considered that there are different freeze‐drying techniques available; however, throughout history, vacuum freeze‐drying or sub‐atmospheric fluidized bed freeze‐drying has been used more frequently (Fang and Bhandari 2012; Gonçalves et al. 2021). It is also important to mention that the main components of freeze‐drying equipment are as follows: (1) refrigeration system, which has the primary function of cooling the shelves of the chamber where the product is located, to freeze the water in the material; (2) product chamber, which is where the product is distributed and dried; (3) vacuum pump, which allows non‐condensable vapors to be eliminated from the system and also generates the low‐pressure condition essential to carry out sublimation; and (4) condenser, which collects the sublimated vapors of the food and condenses them back into solid form as ice (Ishwarya, Anandharamakrishnan, and Stapley 2015; Kılıç, Azak, and Ersus 2024).

The meat industry is one of those that mostly uses freezing; however, it has been shown that incorrect processing affects some quality attributes perceived by consumers. This leads to the search for meat with technological properties closer to refrigerated meat, which minimizes all the changes experienced during freezing–thawing (Kijowski and Richardson 1996). Likewise, it is well known that rapid freezing (cryogenic liquids) offers quality advantages compared to conventional chamber freezing since it influences the size, location (intra‐ or extracellular), and morphology of ice crystals (Arokiyaraj, Dinakarkumar, and Shin 2024; Coria‐Hernández et al. 2020, 2022; Sivarajan et al. 2017).

That is why the main objective of this work is to evaluate the effect of the freeze‐drying process on meat from pork loin (*Longissimus thoracis*) on some quality attributes, such as color, shear force, chemical composition, thermal changes, and modifications in some physicochemical properties of interest, to generate information about the feasibility that could exist for the implementation of this technology at an industrial level as an alternative to conventional processes.

## 2 Materials and Methods

### 2.1 Sample Preparation and Processing Conditions

We worked with castrated male Pietrain pigs of 6 months of age, with an approximate weight of 100 ± 3 kg and 24 ± 1 h *post‐mortem*, coming from a farm with Federal Inspection Type (FIT) certification located in Cuautitlan Izcalli, State of Mexico, Mexico. *Longissimus thoracis* muscle meat with an average weight of 3.7 ± 0.3 kg was obtained from the 9th to 13th rib section and maintained under refrigeration conditions at 4°C ± 2°C, and individual plates of 3 × 3 × 2 cm were cut with a weight of 21.18 ± 0.06 g was analyzed (Coria‐Hernández et al. 2022).

The freezing of the samples was carried out by placing type K thermocouples in the geometric center of each of the samples, and subsequently, they were frozen by indirect contact in liquid nitrogen, whose vapor reached a temperature of −196°C, until the sample reached a temperature indoors of −80°C ± 1°C. Samples were stored in a forced convection chamber Ultima II (Revco, New Castle, DE, USA) at −80°C ± 2°C for up to 5 days.

Some samples were thawed under controlled conditions for subsequent analysis; they were subjected to immersion in water at 35°C for 30 min in a WB05 heating bath (PolyScience, IL, USA) until reaching a thermal center temperature of 20°C ± 1°C.

Subsequently, the samples were lyophilized in a Freezone 4.5 L system (Labconco, KC, USA). The process was carried out with a collector temperature of −49°C ± 1°C for a period of 24 h, under a vacuum pressure inside the chamber of 3.5 ± 0.1 Pa. The lyophilized samples were rehydrated under controlled conditions by immersion in water at 25°C ± 0.5°C and 500 rpm stirring for 2 h to obtain them for subsequent analysis.

The samples analyzed were raw meat without treatment (*R*), frozen–thawed meat (FT), and freeze‐dried‐rehydrated meat (FDR).

### 2.2 Chemical Composition

Chemical analysis was performed according to the methods proposed by the Association of Official Analytical Chemists (AOAC 2023): moisture content (986.21), total ash (990.08), lipids (960.39), and proteins (977.14). For all cases, five replicates were carried out.

### 2.3 Water Activity (*a*_w_), pH, and Water‐Holding Capacity (WHC)

The *a*_w_ value was determined according to the methodology described by van der Sman and Boer (2005) with a 4TE dew point hygrometer (Aqualab, WA, USA) at 25°C ± 1.5°C with five replicates.

For the pH, a meat potentiometer HI99163 (Hanna Instruments, RI, USA) coupled to a stainless‐steel blade was used at 25°C ± 1°C (Krauskopf et al. 2024).

For the WHC, the method described by Barbut (2024) and Szmańko, Lesiów, and Górecka (2021) was used, placing the samples between two 5 × 5 cm absorbent papers and placing them in a press formed by two metal plates. A constant force of 2.5 kg_f_ was applied for 10 min, and at the end, the final weight of the sample and that of the absorbent papers were recorded.

### 2.4 Color Profile Parameters and Surface Myoglobin Fraction

Tri‐stimulus values (L* = lightness, a* = redness, and b* = yellowness) and total color changes (ΔE*) were determined using *R* samples as references. The technique used was reflectance spectrophotometry according to the CIE system (AMSA 2012; Suman and Ramanathan 2024), using a CM600d spectrophotometer (Konica Minolta, Japan) with type A illuminant (incandescent light at 2856 K), a sphere opening of 8 mm, and a 10° viewing angle. The surface myoglobin fraction was determined according to the methodology described by Tang, Faustman, and Hoagland (2004) to find the percentages of deoxymyoglobin (D‐Myo), oxymyoglobin (O‐Myo), and metmyoglobin (M‐Myo).

### 2.5 Shear Force Analysis

A CT3 texture analyzer (Brookfield, MA, USA) was used with a 3 mm flat shear blade (SB geometry) at a test speed of 1 mm/s for 25 s and an activation load of 0.05 kg_f_. The cut was carried out perpendicular to the orientation of the meat fibers at 23°C ± 1°C (AMSA 1995; Coria‐Hernández et al. 2022).

### 2.6 Histological Analysis

1 cm cubes were cut from the central area of the meat after its different treatments. After freezing and lyophilization, 5‐μm‐thick cross sections were cut with the use of a cryostat microtome (CM1950, Leica, Germany). Then the cross sections were stained by the hematoxylin–eosin method (Jin et al. 2023) and rinsed with running water. Histological images were acquired at 40× magnification in a white light field with a Stemi 508 microscope with axiocam color 208 (Carl Zeiss, Oberkochen, Germany). The images were carefully analyzed with Zeiss Zen 3.7 software to determine the changes caused by the different treatments (Dalvi‐Isfahan, Hamdami, and Le‐Bail 2018).

### 2.7 Modulated Differential Scanning Calorimetry (MDSC)

Five replicates per treatment of meat samples (6.0 ± 0.2 mg) were thermally analyzed using a 2920 Temperature Modulated Differential Scanning Calorimeter (TA Instruments, New Castle DE, USA), calibrated at baseline and cell constant with indium (156.6°C) and heat capacity with sapphire at 5°C/min. Nitrogen was used as the purge gas at a constant flow of 60 mL/min with a Refrigeration Cooling System (RCS). The test was carried out from 40°C to 90°C, modulated to ±0.796°C every 60 s. The obtained thermograms were analyzed using TA Instruments Universal Analysis software, 2000 V 4.5 A (New Castle, DE, USA).

### 2.8 Activation Energies

Through the data obtained by MDSC, the activation energies (*E*_a_) required by the proteins during their denaturation were determined. These were obtained using the methodology described by Spigno and De Faveri (2004) to evaluate the initiation of a rearrangement reaction at a structural level, as well as the kinetic behavior of the meat samples to the different treatments.

### 2.9 Experimental Design and Statistical Analysis

The experiment was carried out as a completely randomized design with five replicates. Mean, standard deviation, 1‐way and 2‐way ANOVA, and comparison of means by the Tukey test were performed using the Minitab 16.0.1 software (Penn State University, Pennsylvania, USA). A significance value of *p* < 0.05 was used to identify significant differences between treatments.

## 3 Results and Discussion

### 3.1 Chemical Composition

Chemical composition is important for controlling the main components in meat samples. Table 1 shows the modifications because of the freeze‐drying process on the moisture content, where differences (*p* < 0.05) are observed between the *R* and FDR samples concerning the FT. This is due to various factors, of which the following stand out mainly: (1) the process of formation of large ice crystals during conventional freezing in a chamber, which causes the rupture of some myofibrils, releasing intra‐ and extracellular water, causing an increase in the amount of free water in the form of exudates, decreasing the WHC, results consistent with those presented by Coria‐Hernández et al. (2020) and Hu, Zhang, and Mujumdar (2022); and (2) that the meat samples were not vacuum packed or in modified atmospheres, which caused a phenomenon of ablimation of the relative humidity of the microenvironment during the freezing and storage process, which favored the formation of a thin layer of surface frost on the samples, resulting in higher moisture content values in the FT samples compared to the *R* and FDR (Xia et al. 2009; Arjona‐Román et al. 2017; Nakagawa, Nakabayashi, and Yasunobu 2024). On the other hand, there were no differences (*p* > 0.05) in the results of lipids and total ashes between the analyzed samples.

**TABLE 1:** Results of the chemical composition, *a*_w_, pH, WHC, color profile, surface myoglobin fraction, and shear force of the analyzed samples.

	Sample		
	*R*	FT	FDR
Moisture content (%)	76.71 ± 0.99^a^	79.12 ± 1.66^b^	77.01 ± 0.82^a^
Proteins (%)	20.79 ± 1.16^b^	18.55 ± 0.92^a^	20.56 ± 0.54^b^
Lipids (%)	1.54 ± 0.51^a^	1.49 ± 0.88^a^	1.51 ± 0.08^a^
Total ash (%)	0.96 ± 0.05^a^	0.84 ± 0.06^a^	0.92 ± 0.02^a^
*a*_w_	0.98 ± 0.00^a^	0.99 ± 0.00^a^	0.98 ± 0.01^a^
pH	5.45 ± 0.05^ab^	5.52 ± 0.02^b^	5.44 ± 0.01^a^
WHC (%)	89.23 ± 0.96^c^	82.11 ± 1.25^a^	87.81 ± 0.99^b^
L*	40.12 ± 0.37^a^	42.36 ± 1.03^b^	43.54 ± 0.81^b^
a*	5.89 ± 0.52^b^	4.31 ± 0.67^a^	5.97 ± 0.17^b^
b*	4.97 ± 0.24^a^	7.64 ± 1.04^b^	4.20 ± 0.61^a^
∆E*	—	2.68 ± 0.85^a^	4.95 ± 0.99^b^
Shear force (kg_f_)	9.03 ± 0.38^b^	6.01 ± 0.92^a^	9.57 ± 0.15^b^

*Note:* Mean ± Standard deviation. Means with different letters (a, b, ab,c) in the same row are statistically different (*p* < 0.05).

Proteins play an important role when it comes to meat, being an indicative parameter of most aspects related to some quality attributes such as color and tenderness. In this case, it was found that there are significant differences (*p* < 0.05) in the *R* and FDR samples concerning the FT samples, which is something that agrees with the results obtained by Jo et al. (2022), which indicates that, despite the freeze‐drying conservation process (FDR), the amount of proteins found is very similar to those contained in raw meat without treatment (*R*). On the contrary, samples subjected only to the freezing process lose around 2% of soluble proteins through the exudated liquids, since when there is a rupture at the cellular level of the myofibrils, a possible denaturation by cold and oxidation reactions, among other phenomena. However, many of the proteins such as myoglobin and some sarcoplasmic proteins decrease considerably, which is reflected in various changes at the macromolecular and techno‐functional level, among which stand out changes in the appreciation of color, texture, in the chemical species, and concentrations in which surface myoglobin is found, as reported by Xiufang et al. (2012) and Gap‐Don et al. (2013).

### 3.2 Physicochemical Parameters

In the case of the physicochemical parameters, which are of great importance, Table 1 shows in the first instance the a_w_, a property responsible for indicating the ease for some deterioration reactions to occur in the samples. In this sense, it was found that there are no differences (*p* > 0.05) between the treatments, even though this is related to thermodynamic parameters such as vapor pressure that, indirectly, show the intracellular damage due to the effect of the freezing and/or freeze‐drying (D'Souza and Matthews 2024).

Regarding the pH value, there are differences (*p* < 0.05) between the FT and FDR treatments, indicating that both processes have an important effect on this parameter, favoring the changes that are mainly reflected in the color and the chemical states in which surface myoglobin is found, remembering that pH also plays a fundamental role in the classification of meat as (1) normal, (2) PSE (pale, soft, and exudative), or (3) DFD (dark, firm, and dry) (Jo et al. 2022).

Where there really were important changes (*p* < 0.05) was in the WHC values, which is defined as the ability of meat to bind/maintain water at the cellular level and is closely related to juiciness, pH, and the a_w_. In this sense, according to the process that was subjected, this parameter was decreasing in the FT and FDR samples; this is an indication that there were modifications at the structural level of the proteins, that is, there was a probable denaturation, mainly due to those proteins that are responsible for maintaining water within muscle fibers, such as myosin. In general terms, freezing and lyophilization do have an impact on this parameter, as Jo et al. (2022) mention, with lyophilization (FDR) being the one that allows greater liquid retention compared to conventional freezing (FT).

### 3.3 Color Profile

Regarding the color profile, Table 1 shows the L* coordinate (luminosity) that presents important differences (*p* < 0.05) between the FT and FDR samples concerning the *R* samples; this is the result of several factors, among which the release of exuded liquids due to the effects of the conservation process stands out, which generates a phenomenon called “mirror effect” on the surface of the meat, modifying the optical properties of light reflection, increasing the value of L*, which is closely related to the WHC (Gagaoua et al. 2023). Likewise, it is possible that, since there are structural modifications in the myofibrillar proteins, they also play a fundamental role in the changes that occur in this coordinate.

On the other hand, the a* coordinate (redness) decreases significantly (*p* < 0.05) between the *R* and FDR samples concerning the FT samples. Authors such as Hu, Zhang, and Mujumdar (2022); Jo et al. (2022); and Gagaoua et al. (2023) mention the importance of analyzing this coordinate since its changes are related to changes in the color of the meat, and it is also related to the pH and the chemical states in which the surface myoglobin is found, having a great impact on the decision‐making power of consumers when purchasing meat.

The b* coordinate (yellowness) also suffered important changes due to processing, with a significant increase (*p* < 0.05) in the FT samples, caused by the redox reactions suffered by myoglobin, which are similar to those reported by Gagaoua et al. (2023).

In general terms, there were differences caused by the treatments in the tri‐stimulus coordinates, so when obtaining the total color changes (∆E*), taking fresh meat *R* as a reference, this value is doubled between FT and FDR, indicating that there are changes perceptible to the human eye (Coria‐Hernández et al. 2022) and that in this sense, the consumer could easily distinguish between raw meat, frozen–thawed meat, and meat processed by freeze‐drying‐rehydration.

### 3.4 Surface Myoglobin Fraction

**FIGURE 1:**
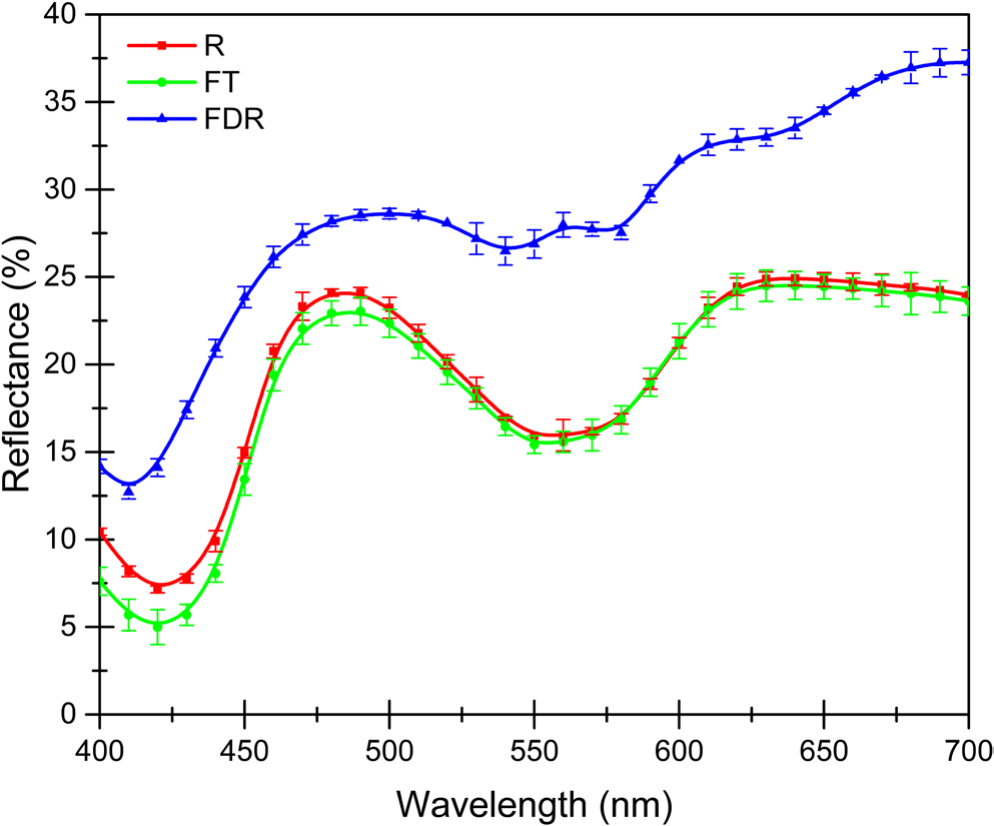
Reflectance spectrum (400 to 700 nm) of the samples with the different treatments.

Figure 1 shows the reflectance curve in the visible light spectrum range (400 to 700 nm), indicating that there are differences (*p* < 0.05) between the different treatments, especially between the *R* and FT samples concerning the FDR samples. These changes are primarily due to the freeze‐drying‐rehydration process, which, as already mentioned, involves modification of the interactions with water since, by generating the sublimation phenomenon and removing water from the meat, it is reincorporated during rehydration and does not interact in the same way or in the same places, as described by Coria‐Hernández et al. (2020).

The area with the most changes occurs between 500 and 600 nm, where there are variations in the slopes of the curves, indicating that there were modifications in the myoglobin on the surface of the samples, either due to redox or oxygenation reactions, considerably modifying the appreciation of the color of the meat (Tang, Faustman, and Hoagland 2004).

**FIGURE 2:**
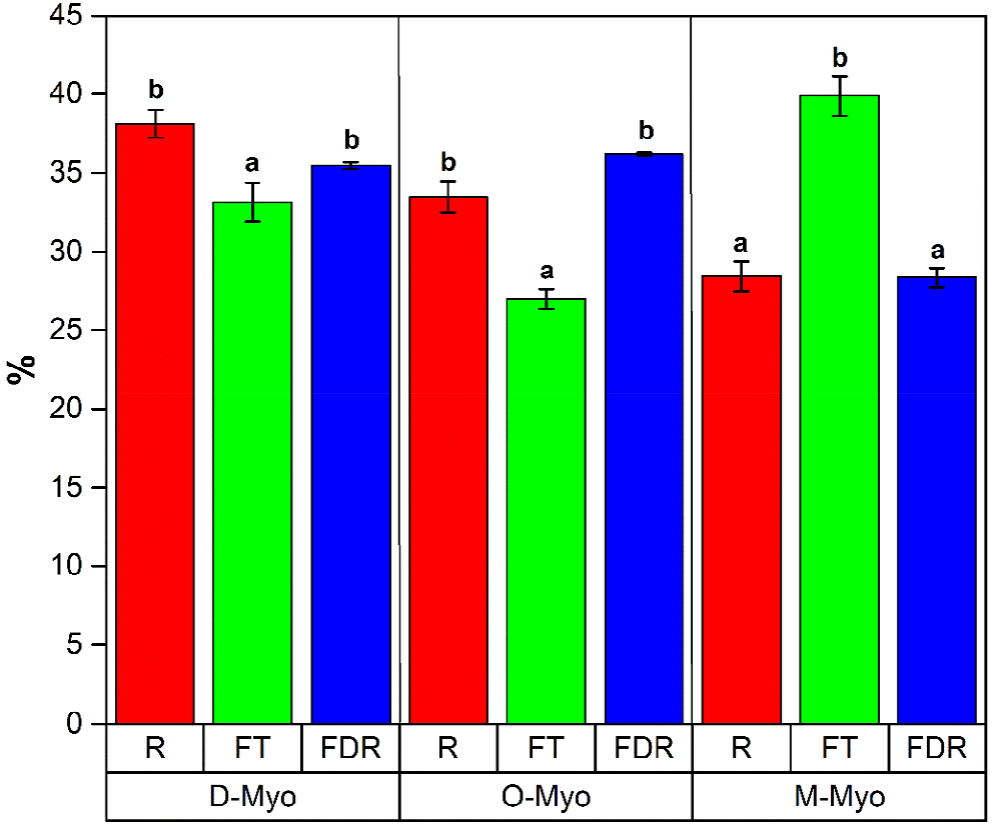
Chemical forms of myoglobin on the surface of meat with different treatments.

Figure 2 represents the percentages of the chemical species of myoglobin in each sample. Gagaoua et al. (2023) and Tang, Faustman, and Hoagland (2004) report that the three main forms of myoglobin always coexist in equilibrium—(1) deoxymyoglobin (D‐Myo), (2) oxymyoglobin (O‐Myo), and (3) metmyoglobin (M‐Myo)—but one of the chemical forms will always predominate, which will be reflected in the changes in its color.

In this sense, in the case of the *R* samples, it was found that the predominant fraction is D‐Myo, which is characteristic of the samples that have gone through an adequate *post‐mortem* biochemical transformation process, resulting in those presented by Jo et al. (2022). However, in the case of the FT samples, the highest concentration obtained is for M‐Myo, providing brown aspects that correspond to what was obtained in the CIELab color profile, indicating that the freezing/thawing process favored the redox reactions on the surface of the meat.

Finally, for the FDR samples, the myoglobin that is found mostly on the surface is O‐Myo, being indicative that the lyophilization‐rehydration process had oxygenation effects of this protein, providing more vivid colorations, results that agree with those obtained in the pH and the tri‐stimulus coordinates (Tang, Faustman, and Hoagland 2004).

### 3.5 Shear Force Test

The shear force is closely related to the tenderness of the meat, and this is a very important quality parameter in the decision of consumers when purchasing meat in supermarkets. For this reason, Table 1 shows the results obtained through the use of mechanical tests, and what was obtained for the *R* samples agrees with the results presented by Kim et al. (2023).

In the case of FT samples, a decrease occurs (*p* < 0.05) compared to raw samples; this occurs due to the effect of the conventional freezing process and the phenomena of intra‐ and extracellular ice crystal formation, which causes denaturation partial protein breakdown and myofibril rupture, causing damage to the structure, causing the shear resistance in the meat to be approximately 33% lower, requiring less effort to be required to generate a cross‐section in the samples.

On the contrary, the FDR samples maintained this parameter very similar (*p* > 0.05) to that of the meat without treatment, indicating that the freeze‐drying‐rehydration process allowed the water that was removed by sublimation and subsequently reincorporated to maintain the mechanical properties of myofibrillar proteins without suffering apparent alterations, as reported by Coria‐Hernández et al. (2022).

### 3.6 Histological Analysis

**FIGURE 3:**
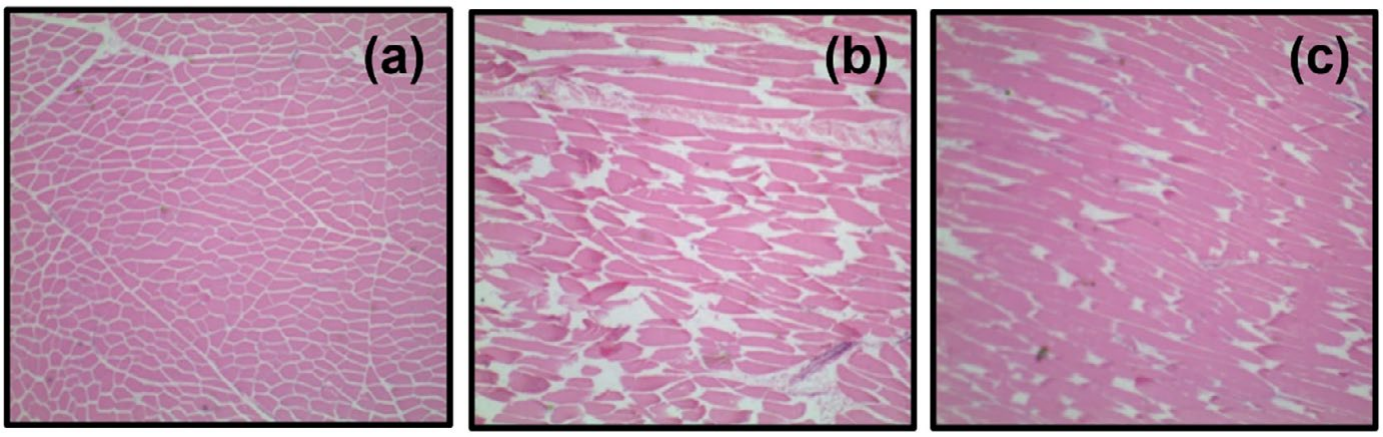
Histological analysis at 40× of the samples with different treatments. (a) *R*; (b) FT; and (c) FDR.

The analysis based on the structural changes that the meat undergoes because of the freezing/thawing and freeze‐drying/rehydration processes is of great importance to relate to the shear force and understand its effect, mainly on the tenderness that the meat can reflect. This is why Figure 3 shows the histological changes of FT and FDR meat compared to meat without *R* treatment, where it is clearly seen that there are important changes at a structural level. In raw meat (Figure 3a), small homogeneous spaces are observed between the myofibrils, where the cellular fibers seem to be intact, maintaining their characteristics and mainly their individuality, which corresponds to studies carried out by Jin et al. (2023) and Wang et al. (2023).

On the other hand, Figure 3b shows the increase in inter‐myofibrillar spaces, showing the existence of damage in the sarcolemma due to the effect of the freezing process and growth of ice crystals, agreeing with the data obtained in the shear force, where this decreased significantly (*p* < 0.05) compared to the *R* samples. The rupture of the perimysium structure may be due to the partial denaturation and greater solubilization of the connective tissue proteins. That is, the perimysium, which represents close to 90% at the intramuscular level, is considered a determining factor in changes in tenderness in meat (Wang et al. 2023).

Regarding the meat subjected to the freeze‐drying process (Figure 3c), it was found that there are also important structural changes at the intercellular level, since, like the FT samples, there is damage to the sarcolemma; however, these are to a lesser extent, indicating that possibly due to the sublimation process of the internal water, it has generated minimal damage to the connective tissue of the meat, agreeing with what was obtained in the shear force tests.

### 3.7 Thermal Analysis by MDSC

**FIGURE 4:**
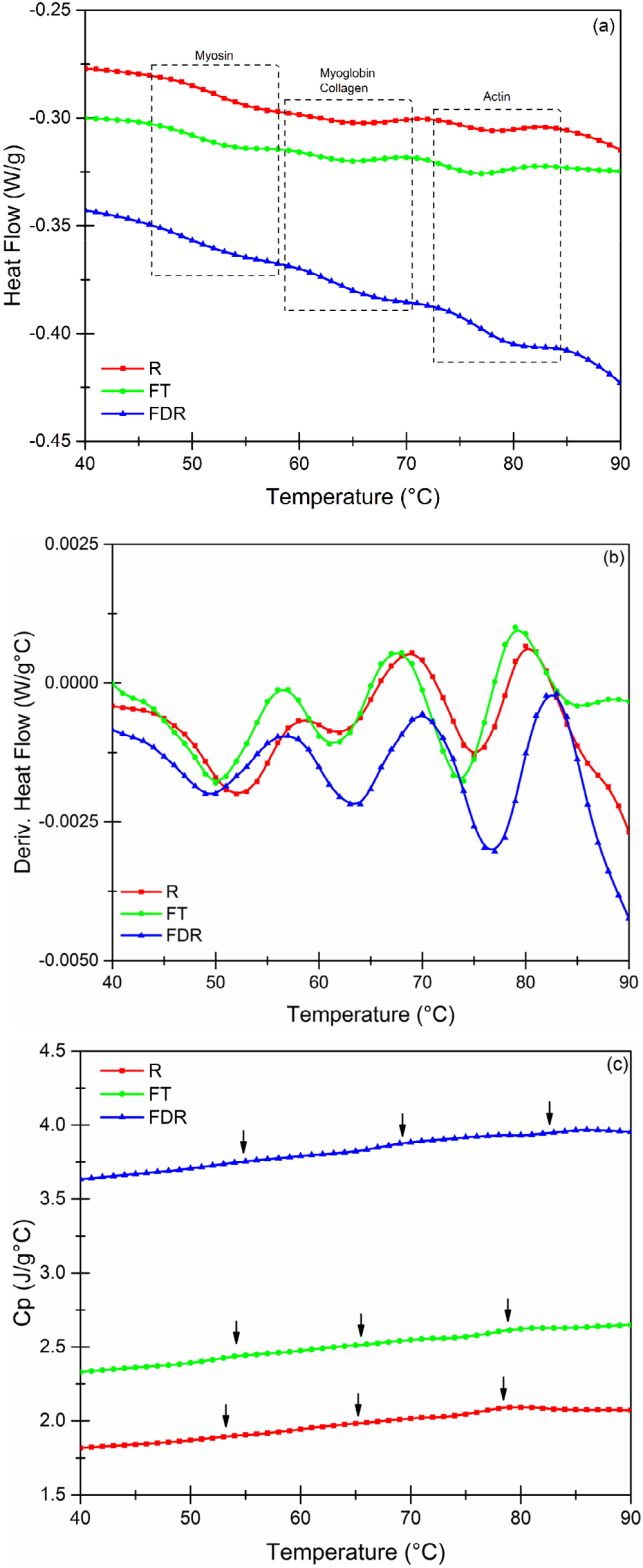
Thermal profiles obtained by MDSC of the samples analyzed under the different treatments. (a) Total heat flow; (b) first derivative of heat flow as a function of temperature; and (c) heat capacity.

About the thermal analysis carried out using MDSC, it was found that the total heat flow (Figure 4a) corresponds to that reported by Visy et al. (2021), clearly showing the three important transition zones in pork loin meat, where the first transition obtained corresponds to the denaturation of myosin (Table 2).

**TABLE 2:** Results of the MDSC analysis of the samples from the different treatments.

	Sample		
	*R*	FT	FDR
Myosin			
*E*_a_ (J/mol)	310.26 ± 2.14^c^	181.51 ± 1.18^a^	292.37 ± 2.26^b^
*T*_D_ (°C)	52.15 ± 0.51^b^	49.32 ± 0.87^a^	49.01 ± 0.67^a^
∆*H*_D_ (J/g)	0.96 ± 0.05^c^	0.12 ± 0.01^a^	0.81 ± 0.02^b^
Myoglobin and collagen			
*E*_a_ (J/mol)	487.75 ± 4.39^b^	345.97 ± 9.34^a^	482.77 ± 3.69^b^
*T*_D_ (°C)	64.11 ± 0.32^a^	62.98 ± 0.88^a^	64.29 ± 0.50^a^
∆*H*_D_ (J/g)	0.71 ± 0.01^a^	0.68 ± 0.07^a^	0.69 ± 0.03^a^
Actin			
*E*_a_ (J/mol)	721.58 ± 5.21^c^	479.33 ± 8.45^a^	679.21 ± 8.27^b^
*T*_D_ (°C)	75.96 ± 1.00^b^	73.01 ± 0.82^a^	77.06 ± 0.55^b^
∆*H*_D_ (J/g)	1.03 ± 0.00^b^	0.71 ± 0.10^a^	0.99 ± 0.04^b^

*Note:* Mean ± Standard deviation. Means with different letters in the same row are statistically different (*p* < 0.05).

Abbreviations: *E*_a_: Activation energy, ∆*H*_D_: Denaturation enthalpy, *T*_D_: Denaturation temperature.

In this case, for myosin (Table 2), the denaturation enthalpy between the samples presented important differences (*p* < 0.05) between them, indicating that there were denaturation phenomena due to the processing effect, with the FT samples being the most affected, despite that there were no differences (*p* > 0.05) between FT and FDR in their denaturation temperatures; so, it is inferred that this is one of the main causes why important effects were found in its structure, which are related to the results obtained in WHC and shear force (Coria‐Hernández et al. 2022).

In this sense, to ensure that the kinetic reactions are being carried out in the proteins of interest, the derivative of the heat flow concerning temperature was obtained (Figure 4b), corroborating the three main energy transitions that correspond to the denaturation of meat proteins.

Regarding activation energies, it must be remembered that these show the minimum amount of energy necessary to start a reaction (Sugakov et al. 2017), in this case in particular, denaturation reactions. Table 2 shows that in the case of myosin and actin, the *E*_a_ are different (*p* < 0.05) between treatments, which response to different causes, among which we highlight that for the FT samples, there is already a partial denaturation due to effect of freezing–thawing and that for samples *R* and FDR, although they are different (*p* < 0.05), there is a lower degree of changes, which is corroborated with the enthalpies and heat capacity.

For myoglobin and collagen, it was found that the *R* and FDR samples are similar (*p* > 0.05) to each other, indicating that the structures of these proteins were not affected by the lyophilization–rehydration process, as reported by Pujol et al. (2023) and agreeing with what was obtained in color profile and shear force.

The second important transition corresponds to myoglobin and collagen, where it was found that there are no differences (*p* > 0.05) between any of the treatments. This could be due to various factors, the first of which would be attributed to the fact that the globular structure of myoglobin and the helical structure of collagen give them a certain degree of thermal stability, reflected not only in the temperatures but also in the denaturation enthalpies.

Regarding actin, it was found that the results obtained in the thermal analysis are very similar to those reported by Visy et al. (2021). In this case, it was found that there are differences (*p* < 0.05) between the *R* and FDR samples for the FT. This may be because the compact and globular structure of actin suffers irreversible damage due to the formation of ice crystals during conventional freezing. However, when it goes through the freeze‐drying process, the damage is not as severe, which means that when the sample is rehydrated, the water that is reincorporated tries to occupy the same empty sites, allowing the protein to try to return to its initial conformation.

Regarding the heat capacity (*C*_p_), it is important to mention that it is the thermal representation of the changes that occur at a structural level that, in this case, corresponds to the denaturation of the main meat proteins (Arjona‐Román et al. 2017). Figure 4c shows the existence (black arrows) of these transitions, and although they are of different magnitude (*p* < 0.05), this allows correlating the effects of the process on the modifications in the structure, allowing the establishment of a structure–function relationship that is reflected in the change of some final quality attributes.

## 4 Conclusions

It was found that the freeze‐drying process has some benefits as a raw meat preservation process compared to conventional freezing, allowing damage to the main meat proteins (myosin, actin, myoglobin, and collagen) because of water sublimation to occur with lower impact, reflected in the maintenance of some sensory quality attributes when rehydrating. This leads to demonstrating the importance of water not only as part of the composition of materials but also in its form of interaction and association at an inter‐ and intramolecular level, thus allowing it to be reflected in macro‐level aspects that are of great relevance for consumers of this type of food.

## Author Contributions

**Jonathan Coria‐Hernández:** conceptualization (equal), formal analysis (equal), methodology (equal), visualization (equal), writing – original draft (equal), writing – review and editing (equal). **Rosalía Meléndez‐Pérez:** conceptualization (equal), methodology (equal), project administration (equal), supervision (equal), validation (equal).

## Ethics Statement

Ethics approval was not required for this research.

## Conflicts of Interest

The authors declare no conflicts of interest.

## Data Availability

Data available on request from the authors.
